# Cerenkov Luminescence Tomography for *In Vivo* Radiopharmaceutical Imaging

**DOI:** 10.1155/2011/641618

**Published:** 2011-04-07

**Authors:** Jianghong Zhong, Chenghu Qin, Xin Yang, Shuping Zhu, Xing Zhang, Jie Tian

**Affiliations:** Intelligent Medical Research Center, Institute of Automation, Chinese Academy of Sciences, Beijing 100190, China

## Abstract

Cerenkov luminescence imaging (CLI) is a cost-effective molecular imaging tool for biomedical applications of radiotracers. The introduction of Cerenkov luminescence tomography (CLT) relative to planar CLI can be compared to the development of X-ray CT based on radiography. With CLT, quantitative and localized analysis of a radiopharmaceutical distribution becomes feasible. In this contribution, a feasibility study of *in vivo* radiopharmaceutical imaging in heterogeneous medium is presented. Coupled with a multimodal *in vivo* imaging system, this CLT reconstruction method allows precise anatomical registration of the positron probe in heterogeneous tissues and facilitates the more widespread application of radiotracers. Source distribution inside the small animal is obtained from CLT reconstruction. The experimental results demonstrated that CLT can be employed as an available *in vivo* tomographic imaging of charged particle emitters in a heterogeneous medium.

## 1. Introduction

Optical molecular imaging is a typical multidisciplinary method promoted by biological, physical and chemical sciences [1]. It is a powerful biomedical research tool [2] with the advantage of having a low cost. However, there are significant challenges for commercialization of optical imaging probes, especially for clinical studies [3]. To our best knowledge, only one optical molecular imaging probe approved by the US Food and Drug Administration (FDA), indocyanine green (ICG), is used in clinical studies. Recently, on the utilization of the Vavilov-Cerenkov effect, Liu et al. [4] demonstrated the quantitative analysis of both radioactive optical images and positron emission tomography (PET) or single photon emission computed tomography (SPECT) images of living subjects with a wide diversity of radioactive probes. 

Cerenkov luminescence is emitted from Cerenkov radiation [5] during the initial decay process of medical isotopes. These Cerenkov photons are detected with a low-noise charge-coupled device (CCD) and converted into optical images, which is Cerenkov luminescence imaging (CLI) [6]. As an optical molecular imaging method, CLI can quantitatively map [7] the distribution of radionuclides, such as ^18^F, ^131^I, and ^225^Ac. 

In order to obtain spatial and/or temporal localization information of the radioactive source inside the medium, Li et al. [8] proposed the corresponding Cerenkov luminescence tomography (CLT), assuming that the scattering and absorption properties of tissues were homogeneous. However, small animal inherently has optical heterogeneity [9]. It is necessary to improve the physical model so as to more accurately visualize the *in vivo* radioactive probe distribution.

As for the meaning of optical coefficients *μ*_*a*_(*r*) and *μ*_*s*_(*r*), we can refer to the following definition [10]: *μ*_*a*_(*r*) is defined as the probability of photon absorption in a medium per unit path length, of which the reciprocal is referred to as the mean absorption length; *μ*_*s*_(*r*) is defined as the probability of photon scattering in a medium per unit path length, of which the reciprocal is referred to as the scattering mean free path. We usually describe the scattering property of biological tissue as *μ*_*s*_′(*r*) = (1 − *g*)*μ*_*s*_(*r*), which is the reduced scattering coefficient.

Our aim in this paper is to describe a feasibility study for CLT in *in vivo* radiopharmaceutical imaging. The mathematical model [10] of the proposed method is the diffusion approximation. Although Lv et al. [11] evaluated the performance of the algorithm on the heterogeneous phantom for bioluminescence tomography (BLT), its performance in the inverse problem of CLT still requires further study. The model-based iterative reconstruction is applied to *in vivo* radioactive optical imaging in the paper. The results of the physical experiments on nude mice demonstrated that the proposed technique with *a priori* structural information incorporated in the CLT inverse problem can improve the quality of source reconstruction.

## 2. Methods

### 2.1. Diffusion Model

The *in vivo* Cerenkov light emission spectrum [6] is quite wide (400–800 nm) and overlaps with the so-called near infrared window of biological tissues, when light scattering dominates light absorption. This photon propagation can be modelled by the steady-state diffusion equation (DE) and Robin boundary condition [12]. After a series of transformations, the matrix form of DE on the discretized mesh with the permissible source region strategy [13] can be obtained. It describes the linear relationship between the boundary measured photon flux density Φ and the unknown source density in the permissible source region *S* as follows:

(1) AS=Φ,

where *A* is a matrix generated from the DE model.

### 2.2. Image Reconstruction and Fusion

Because the inverse problem is ill-posed for (1), we applied the method of Tikhonov regularization. The reconstruction stability and convergence were illustrated through numerical studies on the heterogeneous phantom [11]. In the paper, the method of the image fusion was published in Zhu's Ph.D. thesis with mathematical proof [14].

### 2.3. Materials and Instruments

The female Nu/Nu nude mice used in the experiments were purchased from the Department of Laboratory Animal Science, Peking University Health Science Centre. FDG was kindly provided by the Department of Nuclear Medicine, Beijing Union Medical College Hospital. Animal experimentation was conducted under approved research protocols of the Animal Care and Use Committee. 

**Figure 1:**
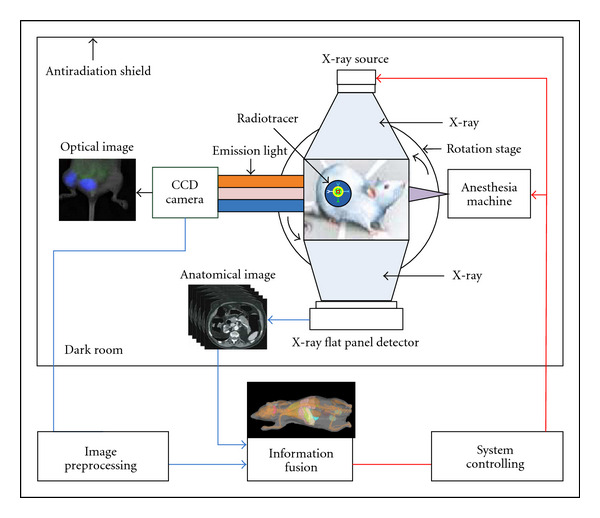
Schematic diagram of our imaging system. *In vivo* imaging system consists of a CCD camera, small animal anesthesia machine, a Micro-focus X-ray source, an X-ray flat panel detector, a rotation stage, and an antiradiation shield.

All imaging experiments were performed with an *in vivo* molecular imaging system developed by our group, as shown in Figure 1. The CCD camera (Princeton Instruments VersArray 1300 B, Roper Scientific, Trenton, NJ) has 1340 × 1300 pixels with 20 × 20 *μ*m-sized pixels. The typical work temperature of the CCD chip is −110°C. Low readout and binning noise makes this camera ideal for Cerenkov optical imaging. The optical imaging system has a dark room that can block both all external lights and internal high-energy radiation. There is a local expansion and statistics algorithm that can remove the noise of the Cerenkov optical images prior to CLT reconstruction [15]. Meanwhile, the camera was calibrated with an integrating sphere (USS-1200 V-LL Low-Light Uniform Source, Labsphere, North Sutton, NH). The entire optical system was used for data acquisition of Cerenkov luminescent images. The final quantitative calibration formula for the optical system is given by

(2) $$S\left(r\right)=\left(\frac{2.09p}{t}+10.89\right)\times 10^{-10}\;\text{W}\cdot {\text{mm}}^{-2},$$

where *p* is the CCD pixel intensity value and *t* is the exposure time with *s* units. The Micro-CT system provided 3D anatomical information in accordance with normal usage [16]. The small animal bed was marked with 24 simultaneously identifiable markers by the optical and Micro-CT systems. System control and image processing were carried out on a personal computer with Intel Core(TM)2 Duo Processor 2.33 GHz and 2 GB RAM.

### 2.4. Radioactive Optical Imaging

**Figure 2:**
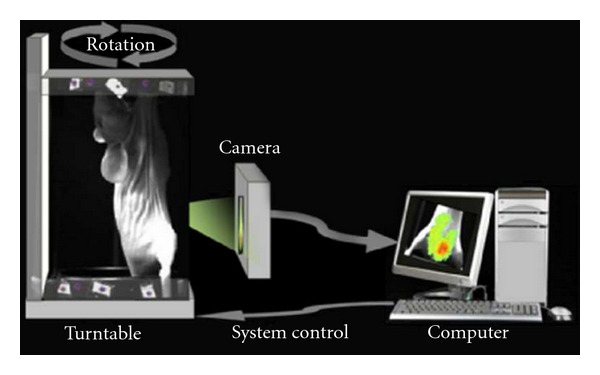
System block diagram of the *in vivo* imaging system, developed by ourselves. The region near the bladder belongs to the view of the CCD camera.

In each experiment, we needed to rotate the turntable 360 degrees to have access to four luminescent images and the corresponding photograph images (Figure 2). A CLI can be obtained after the overlay process between the luminescence image and the corresponding photograph image.

### 2.5. Structural and Optical Information Fusion

The dimension of the reconstructed Micro-CT data was 400 × 400 × 560. The voxel size of the Micro-CT data was 0.15 × 0.15 × 0.15 mm. The Micro-CT data and four CLI images were registered using fixed markers. 3D Micro-CT data was discredited into a tetrahedral mesh. 2D photos were then mapped onto the surface of the mesh according to the registration relation. The 3D finite element mesh with the Cerenkov light intensity on the surface was the output of the integration of structural and optical information.

### 2.6. Parameters of CLT Reconstruction

The entire finite element framework and the Tikhonov regularization method were implemented in C++ code. All of the parameters of the finite element framework were the same as those used in [11], except for the optical properties. The regularization parameter was set to 0.01. In order to analyze the algorithm more reasonably, the DisError is defined as the distance error of the distance between the actual source position and the reconstructed source position by the *L*_2_ norm as follows:

(3) $$\text{DisError}={||r_{1}-r_{2}||}^{2},$$

where *r*_1_ is the central coordinate of the reconstruction source with maximum density and *r*_2_ is that of the actual source center.

## 3. Results

**Figure 3:**
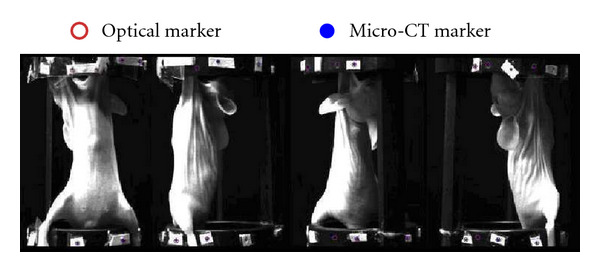
The views of the mouse bed with the fixed markers. The healthy mouse was injected with Fenestra LC and FDG. The rotation stage is set to rotate at 0°, 90^°,^ 180°, and 270° for taking photos respectively. The CCD camera was fixed in front of the mouse bed.

A 21 g mouse was anesthetized with 2% isoflurane delivered in medical air. It was successively injected with 0.20 ml of Fenestra LC and 11.10 MBq of 2′-deoxy-2′-[^18^F]fluoro-D-glucose (FDG) via the tail vein. The healthy mouse was intactly affixed on the small animal bed, as shown in Figure 3. After half an hour, the mouse was scanned by the Micro-CT system, and the system independent parameters of power, voltage, and exposure time were set at 50 W, 50 kVp, and 0.47 s, respectively. After waiting for a quarter of an hour, we used the camera to collect the Cerenkov luminescence images with the aperture number f to 2.8, the binning value equal to 2 and the integration time equal to 180 s. There was no optical filter, because the Cerenkov light signal was very weak. A CLI was obtained after a 90-degree rotation of the turntable, as shown in Figure 3.

### 3.1. CLT Reconstruction

**Figure 4:**
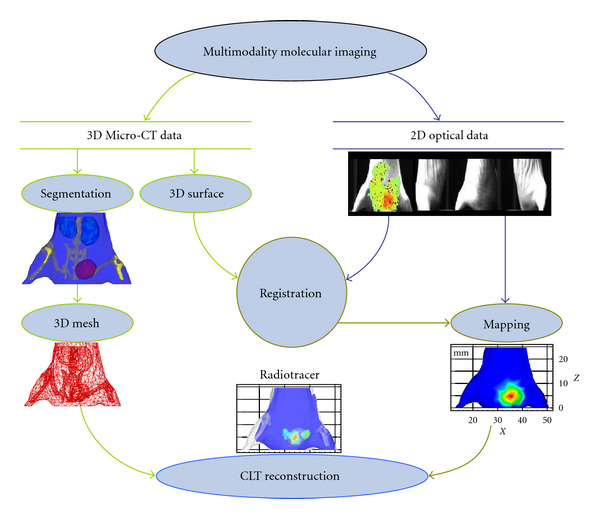
Schematic of radioactive source distribution reconstruction with optical property heterogeneity. The mouse was scanned by our multimodal molecular imaging system. 3D MicroCT data and planar optical images were collected by the same system. The interval degree was 90 for multiangle imaging. 2D optical intensity distribution is mapped on the 3D surface in accordance with the registering relationship. CLT is the output of the information fusion and optical tomography reconstruction.

The designed CLT reconstruction process with optical property priors is illustrated in Figure 4. During the processing, there was a relatively concentrated and strong Cerenkov light near the bladder region. The geometric center (34.70 mm, 14.49 mm, 5.13 mm) of the bladder was chosen as the actual light center according to the acquired Micro-CT information. Physically, the bladder is filled with liquids. FDG uptake and distribution, as a liquid in the bladder, should be homogeneous. We selected part of the reconstructed Micro-CT data along the *Z* axis from the 65th to the 229th slice to reconstruct the source position. The segmented Micro-CT data with the dimension equal to 400 × 400 × 165 was discretized into 3555 points, 38115 triangles and 18690 tetrahedrons. Figure 4 shows the surface of tissues including muscle, kidneys, bladder, and bone. The volume percentage of each tissue is 77.14% (muscle), 3.27% (kidneys), 2.27% (bladder), and 17.32% (bone). The 2D photos were then mapped onto the surface of the mesh. The mesh and Cerenkov light intensity distribution on the surface were used for CLT reconstruction. The optical parameters of the mouse [9] are shown in Table 1. Here, *μ*_*a*_(*r*) is the absorption coefficient; *μ*_*s*_′(*r*) is the reduced scattering coefficient. These were the weighted values because of the mixed optical spectrum used in the experiment. According to the surface light distribution (Figure 4), the permissible source region was set as follows:

(4)   P={r ∣ 30<x<40 mm,  11<y<20 mm, 2<z<8 mm}.

Finally, we carried out the reconstruction. In theory, it is an optimization problem to compute source distribution, which seeks a regularized difference minimizing the observed boundary measurements of light distribution and the boundary measurements predicted from a mathematical model.

### 3.2. CLT with and without Optical Property Priors

**Figure 5:**
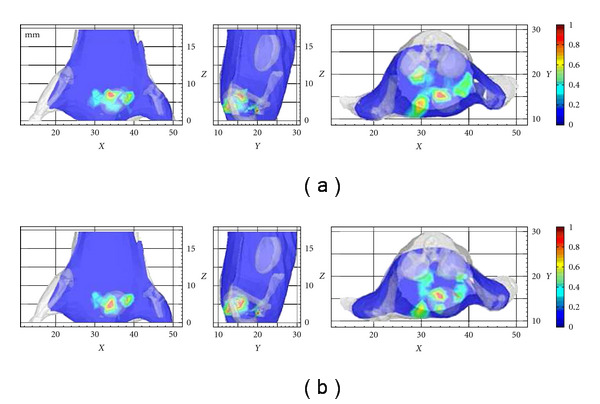
Slices of the *in vivo* flux density distribution through the reconstructed source center successively from *XZ*, *YZ*, and *XY* views. (a) Three slices from 3D views in HM; (b) three slices from 3D views in HR. Peak values of the flux densities in different slices were normalized to 1, in order to simultaneously demonstrate reconstructed source centers. The unit of the *X*, *Y*, and *Z* axes is in mm.

The proposed technique on a homogeneous model (HM) and heterogeneous model (HR) were performed. In the HM experiment, the optical property parameters of the mouse in the diffusion model were set to *μ*_*a*_(*r*) = 4.10 × 10^−2^ mm and *μ*_*s*_′(*r*) = 67.27 × 10^−2^ mm in terms of the volume percentage, because there were no optical property priors. Matrix A formed in the reconstruction procedure was 737 × 1371. Next, we performed the CLT reconstruction with the optical property priors. This was the HR experiment. We could obtain two sets of the reconstructed results, as shown in Figure 5. The center of the tetrahedron with the maximum intensity was used as the reconstructed source center (RSC.). The quantitative information of the results from both HM and HR is summarized in Table 2. The reconstruction times of the proposed method in the last reconstruction procedure were only 0.30 s. Based on the HR, the distance error was only 1.41 mm, but was 2.32 mm in the HM after inverse reconstruction. These results proved that optical property heterogeneity can help CLT reconstruction.

## 4. Discussion and Conclusion

CLT simultaneously has the characteristics of radioisotope-labeled molecules and optical imaging. Coupled with the information on the anatomy of the small animal, the reconstructed distance error was reduced. These encouraging results proved that optical property heterogeneity can help CLT reconstruction. Furthermore, source depth and flux density distribution in the mouse were calculated by the rule of CLT reconstruction. This paper proposed a theoretical approach to the pharmacokinetic and dynamic imaging for radioactive probes in different tissues.

More research is needed to improve the spatial resolution of the inverse source reconstruction. It is necessary to further explore the laws of Cerenkov light propagation in small living animals. This may give us a more practical mathematical model, such as multispectral optics, general approximation, and whole body reconstruction. We should improve the *in vivo* optical imaging system and explore the best optimization time of the radioactive tracer drug injection and image acquisition in order to adapt to a lower dose of radioisotopes. The paradox is that we need to detect enough light to carry out the CLT reconstruction. For a tumor model, the specificity, dose of the radionuclide, and the tumor cell characteristics will impact the minimum concentration of the radiotracer for CLT reconstruction. In addition, the anatomical location and shape of tissues or tumors to be recovered needs to be reconsidered, such as determining their depth.

The linear relationship between Cerenkov optical images and PET/SPECT images has been accepted as common knowledge. In theory, Cerenkov radiation is produced during the initial decay process, and is thereby more localized to the decay event than the annihilation event tracked by PET/SPECT scanning. The Cerenkov radiation spectrum is weighted toward the ultraviolet and blue bands. Optical absorption and scattering will reduce the sensitivity of CLT. However, one advantage of CLT is that it is a low cost imaging system and is known for its ease of operation.

In conclusion, the distribution of the radiopharmaceutical inside the heterogeneous medium can be imaged by using the proposed CLT technique without PET/SPECT. This will allow the spread of *in vivo* pharmacokinetic imaging research with a certain radiotracer to laboratories with limited budgets.

## Figures and Tables

**Table 1:** Optical parameters of the nude mouse [17], 10^−2^ mm^−1^.

Material	*μ*_*a*_(*r*)	*μ*_*s*_′(*r*)
Muscle	3.20	58.60
Kidneys	1.00	83.00
Bladder [18]	68.40	139.00
Bone	0.24	93.50

**Table 2:** Comparison of CLT with and without optical property priors.

Model	HM.	HR.
Maximum density (10^−10^ W·mm^−2^)	12.84	12.98
RSC. (mm)	(34.12, 15.67, 6.93)	(34.12, 15.67, 6.93)
DisError (mm)	2.23	1.41
